# *Ngn3* Regulates Differentiation Competence of Retinal Progenitor Cells Through Transcriptional and Epigenetic Modification

**DOI:** 10.3390/ijms27093845

**Published:** 2026-04-26

**Authors:** Canbin Chen, Huilin Liang, Qinghai He, Shuyi Chen

**Affiliations:** State Key Laboratory of Ophthalmology, Guangdong Provincial Key Laboratory of Ophthalmology and Visual Science, Zhongshan Ophthalmic Center, Sun Yat-sen University, Guangzhou 510060, China; canbin_chen@outlook.com (C.C.); lianghuilin1567@163.com (H.L.); hqh2025@outlook.com (Q.H.)

**Keywords:** *Ngn3*, retinal progenitor cell, retinal development, cell fate reprogramming, transcriptome, chromatin accessibility

## Abstract

The retina is a complex sensory neural tissue composed of six major types of neurons and one type of glial cell. The cell fate specification of retinal cells is tightly governed by intrinsic factors and extrinsic microenvironmental cues. Among the key regulators directing retinal cell fate differentiation is a group of bHLH family transcription factors (TFs). Our previous work demonstrated that the bHLH TF *Ngn3* exhibits robust potential to induce retinogenesis in both distantly related fibroblasts in vitro and late retinal progenitor cells (RPCs) in vivo. However, the underlying molecular mechanisms remain largely elusive. In this study, we combined immunohistological examination and RNA-seq and ATAC-seq analyses to investigate the cellular and molecular mechanisms governing *Ngn3*-driven retinogenesis in late RPCs. Our results revealed that *Ngn3* overexpression promotes premature cell cycle exit in late RPCs and remodels their transcriptomic and epigenomic landscape towards a state favoring rod photoreceptor and RGC differentiation. Furthermore, cross-comparison with *Ngn3*-overexpressing fibroblasts in vitro revealed cell-type-specific mechanisms underlying *Ngn3*-mediated neuronal fate reprogramming. These findings advance our understanding of Ngn family-mediated retinal cell fate regulation and provide a mechanistic framework for optimizing *Ngn3*-based retinal regeneration strategies for the treatment of retinal degeneration diseases.

## 1. Introduction

The retina is a complex sensory neural tissue composed of six types of neurons—retinal ganglion cells (RGCs), amacrine cells, bipolar cells, horizontal cells, rod photoreceptors, and cone photoreceptors—and one type of glial cell, Müller cells [1,2]. All these retinal cell lineages are generated by multipotent retinal progenitor cells (RPCs) during development in a sequential yet overlapping manner. Throughout this process, RPCs progressively modulate their differentiation capacity, the so-called ‘competent states’, to generate distinct retinal cell types at different developmental stages. For instance, in mice, early RPCs in embryonic retinas exclusively give rise to early-born retinal cell types, such as RGCs and cones, whereas late RPCs in postnatal retinas only produce late-born retinal lineages, including rod photoreceptors, bipolar cells, and Müller glial cells [3,4,5].

The competent states of RPCs, as well as the fate commitment and differentiation of all retinal cell types, are tightly controlled by intrinsic regulators and extrinsic microenvironmental cues. Among these regulatory factors, transcription factors (TFs)—particularly those belonging to the basic helix–loop–helix (bHLH) family and homeodomain family—play pivotal roles [6,7,8,9]. For example, the homeodomain TF *Pax6* is highly expressed in RPCs and governs their proliferation and multipotency [10]; the bHLH family TF *Atoh7* is expressed in early RPCs and directs their commitment to the RGC differentiation pathway [11,12]; downstream of *Atoh7*, Pou family of homeodomain TFs promote the terminational differentiation and maturation of RGCs [13]; while rod photoreceptor fate specification is strictly controlled by *Otx2* and *Crx* [14,15]. A comprehensive elucidation of the molecular mechanisms by which TFs drive retinal cell fate determination is not only a central question in developmental biology but also a fundamental prerequisite for developing innovative strategies to regenerate retinal neurons for the treatment of retinal degeneration diseases. The Neurogenin (Ngn) family members are potent neurogenic bHLH-domain TFs consisting of three members: *Ngn1*, *Ngn2*, and *Ngn3* [16]. These TFs are widely expressed in the central and peripheral nervous system from the early embryonic stage [17], and play critical roles in directing the fate differentiation of specific neuronal subtypes [16,18,19,20]. In the mammalian retina, only *Ngn2* is endogenously expressed [17], with its expression initiating in RPCs at the earliest stages of retinogenesis and controlling the leading edge of the retinogenesis [21]. In contrast, *Ngn1* and *Ngn3* are not detected in the developing retina [17]. Beyond development, recent studies have demonstrated that overexpression of all three Ngn family members, in combination with other TFs, efficiently induces the transdifferentiation of distantly related fibroblasts into functional neurons [22,23,24]. This highlights the robust neurogenic capacity of Ngn family TFs in direct somatic cell reprogramming and identifies them as ideal candidates for neural fate reprogramming in therapeutic applications.

Inspired by the studies described above, our previous work investigated the ability of Ngn family TFs to reprogram fibroblasts into retinal neurons, revealing that all three members alone can induce RGC-like neuron fate in fibroblasts [25]. Notably, although only *Ngn2* is endogenously expressed and functional in the retina, *Ngn1* and *Ngn3* exhibit stronger RGC fate-inducing activity than *Ngn2* in fibroblast reprogramming. More intriguingly, we found that ectopic expression of *Ngn1* and *Ngn3* in late RPCs alters their differentiation competence: it promotes rod photoreceptor and RGC fates while repressing Müller glial cell and bipolar and amacrine interneuron fates [25]. These observations suggest that Ngn family TFs may serve as in vivo reprogramming tools to regenerate retinal neurons for the treatment of retinal degenerative diseases. Despite these well-established phenotypic observations from our prior study, the underlying genome-wide transcriptional and epigenomic mechanisms whereby Ngns remodel the intrinsic fate potential of late RPCs remain largely uncharacterized. In this study, we integrate immunohistological examination and RNA-seq transcriptomic and ATAC-seq epigenomic analyses to investigate the cellular and molecular mechanisms governing *Ngn*-driven reprogramming of the differentiation competence states in late RPCs. Given the relatively stronger capacity of *Ngn3* in directing retinal neuron fate reprogramming in both in vitro and in vivo systems compared with *Ngn1* and *Ngn2* [25], and to minimize confounding effects from endogenously expressed retinal factors, we focus on *Ngn3* in the present study. The findings of this study will advance our understanding of Ngn family-mediated retinal cell fate regulation and provide a mechanistic framework for optimizing *Ngn3*-based retinal regeneration strategies.

## 2. Results

### 2.1. Ngn3 Drives RPCs to Exit the Cell Cycle

**Figure 1:**
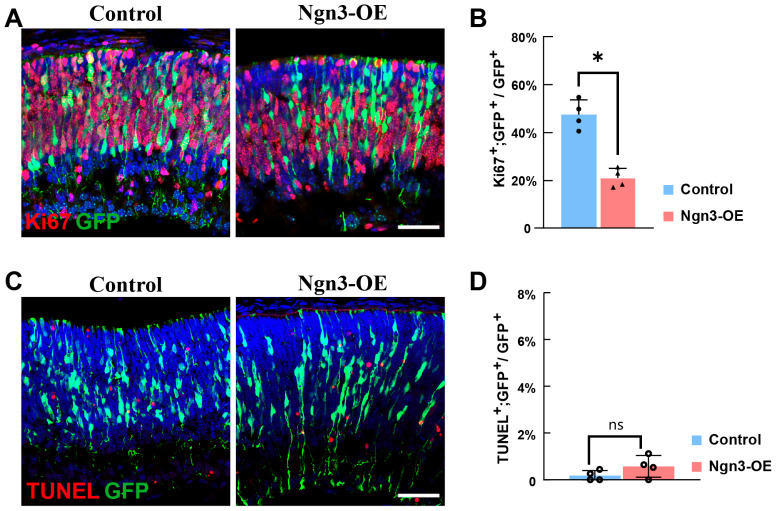
*Ngn3* drives RPCs to exit the cell cycle. (**A**) Confocal immunofluorescent staining images of Ki67 and GFP in retinal cryosections from Control and *Ngn3*-OE groups at DAE 2, GFP labels electroporated late RPCs (**B**) Quantification of the percentage of Ki67^+^; GFP^+^ cells among total GFP^+^ electroporated RPCs in Control and *Ngn3*-OE groups. (**C**) Confocal immunofluorescent staining images of TUNEL assay of retinal cryosections from Control and *Ngn3*-OE groups at DAE 2, GFP labels electroporated late RPCs. (**D**) Quantification of the percentage of TUNEL^+^; GFP^+^ cells among total GFP^+^ electroporated RPCs in Control and *Ngn3*-OE groups. Data are presented as mean ± standard deviation (SD). Statistical significance was determined by an unpaired two-tailed *t*-test. ns, not significant; * *p* < 0.05. n ≥ 3 independent experiments. Scale bar: 50 μm.

Our previous work demonstrated that *Ngn3* overexpression (*Ngn3*-OE) in late RPCs promotes rod photoreceptor fate differentiation while inhibiting Müller glial cell and bipolar and amacrine interneuron differentiation [25]. To explore the mechanisms by which *Ngn3* modulates retinal cell fate differentiation in late RPCs, we first assessed the proliferation activity of *Ngn3*-OE late RPCs, as progenitor cell differentiation is tightly coupled to proliferation. For this purpose, we performed in vivo electroporation on newborn mouse pups to introduce *Ngn3*-OE plasmids into late RPCs, and collected the retinas at day 2 after electroporation (DAE 2). We then performed Ki67 immunofluorescent staining on the retinal cryosections to evaluate the proliferation status of the retinal cells. The results showed that, in control retinas, 49.73 ± 4.94% of GFP^+^ electroporated RPCs exhibited Ki67 signals, indicating active proliferation (Figure 1A(left),B). In contrast, in *Ngn3*-OE retinas, the proportion of Ki67^+^ actively proliferating cells was significantly reduced to 20.95 ± 3.95% (Figure 1A(right),B). These results demonstrated that *Ngn3*-OE induces premature cell cycle exit in late RPCs.

We next examined whether *Ngn3*-OE affects the survival of late RPCs. To this end, we performed Terminal deoxynucleotidyl transferase dUTP nick end labeling (TUNEL) assay on DAE 2 retinas. Sporadic apoptotic events were observed in the developing retina at this stage; however, GFP-labeled electroporated RPCs rarely underwent apoptosis in the control group (Figure 1C(left),D), and *Ngn3*-OE did not exert a significant effect on the survival of late RPCs (Figure 1C(right),D). These results demonstrated that *Ngn3*-OE does not affect the survival of late RPCs.

### 2.2. Ngn3-OE Profoundly Remodels the Transcriptome of Late RPCs

**Figure 2:**
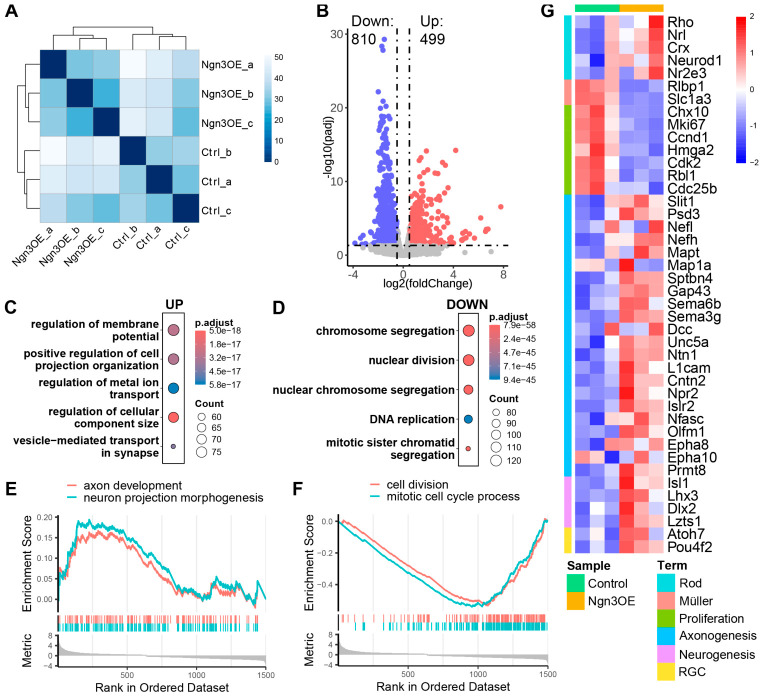
Transcriptomic profiling of *Ngn3*-OE RPCs via RNA-seq. (**A**) Heatmap showing sample correlation scores derived from RNA-seq data of control and *Ngn3*-OE late RPCs at DAE 2. (**B**) Volcano plot showing differentially expressed genes (DEGs) between *Ngn3*-OE and control late RPCs. Red dots represent upregulated genes, and blue dots represent downregulated genes. DEGs are defined as genes with log_2_(fold change) ≥ 0.5 or ≤−0.5 and adjusted *p*-value ≤ 0.05. (**C**,**D**) Bubble plots showing the top five significantly enriched Gene Ontology (GO) terms for upregulated (**C**) and downregulated (**D**) DEGs in *Ngn3*-OE late RPCs compared to control late RPCs. (**E**,**F**) Gene Set Enrichment Analysis (GSEA) plots showing pathways enriched among upregulated (**E**) and downregulated (**F**) DEGs. (**E**) neuron projection development (NES = 0.939, Padj = 0.950) and axon development (NES = 0.921, Padj = 0.963). (**F**) cell division (NES = −3.058, Padj = 0.085) and mitotic cell cycle process (NES = −3.208, Padj = 0.085). (**G**) Heatmap showing the expression levels of representative genes in control and *Ngn3*-OE samples.

As a TF, *Ngn3* exerts its cellular functions primarily by regulating the transcription of target genes. To elucidate the molecular mechanisms underlying *Ngn3*-OE-mediated reprogramming of late RPC differentiation competence, we performed RNA-seq on electroporated late RPCs. To this end, we collected GFP-labeled control and *Ngn3*-OE electroporated late RPCs at DAE 2 via fluorescence-activated cell sorting (FACS), and performed RNA-seq analyses. Sample correlation distance analysis revealed close clustering of biological replicates within each experimental group, validating the quality of the RNA-seq data, while control and *Ngn3*-OE samples formed distinct clusters, suggesting that *Ngn3*-OE profoundly altered the transcriptome of late RPCs (Figure 2A). We then performed Differentially Expressed Gene (DEG) analyses to search for genes whose expression changed upon *Ngn3*-OE in late RPCs. The results showed that, at the threshold set at a log_2_ (fold change) ≥ 0.5/≤ −0.5 and an adjusted *p* value ≤ 0.05, 499 genes were upregulated and 810 genes were downregulated in *Ngn3*-OE late RPCs compared with control RPCs (Figure 2B, and Supplementary Material S1). To examine what cellular processes were most significantly affected by *Ngn3*-OE in late RPCs, we performed gene ontology (GO) term enrichment analyses and gene set enrichment analysis (GSEA) analyses. The results showed that the upregulated gene set was significantly enriched for cellular processes related to neuronal function, such as ‘regulation of membrane potential’, ‘positive regulation of cell projection organization’, and ‘vesicle-mediated transport in synapse’ (Figure 2C,E). In addition, inspecting specific genes key to the cell fate development of retinal cells showed that several key rod photoreceptor fate determination TFs and functional genes, such as *Rho*, *Nrl*, *Crx*, and *Nr2e3*, and several key TFs involved in RGC fate development TFs, such as *Atoh7* and *Pou4f2* were upregulated in *Ngn3*-OE late RPCs (Figure 2G). These findings were consistent with the promotion of rod photoreceptor and RGC lineages observed at DAE 14 in our previous study [25], indicating that the early transcriptomic changes at DAE 2 are associated with subsequent cell fate commitment. On the other hand, the downregulated gene set was highly enriched for cell cycle progression processes, such as ‘chromosome segregation’, ‘nuclear division’, and ‘DNA replication’ (Figure 2D,F). This enrichment directly reinforces the premature cell cycle exit phenotype observed above (Figure 1). Additionally, key Müller glial cell functional marker genes, such as *Rlbp1* and *Slc1a3*, were downregulated in *Ngn3*-OE late RPCs, which is consistent with the repressed Müller fate observed at DAE 14 in our previous study [25].

Collectively, RNA-seq analyses demonstrated that *Ngn3*-OE induces extensive transcriptomic remodeling in late RPCs, which is correlated with the observed premature cell cycle exit and altered retinal cell differentiation competence.

### 2.3. Ngn3 Reprograms Distinct Somatic Cell Types via Divergent Molecular Pathways with a Conserved Neurogenic Core

**Figure 3:**
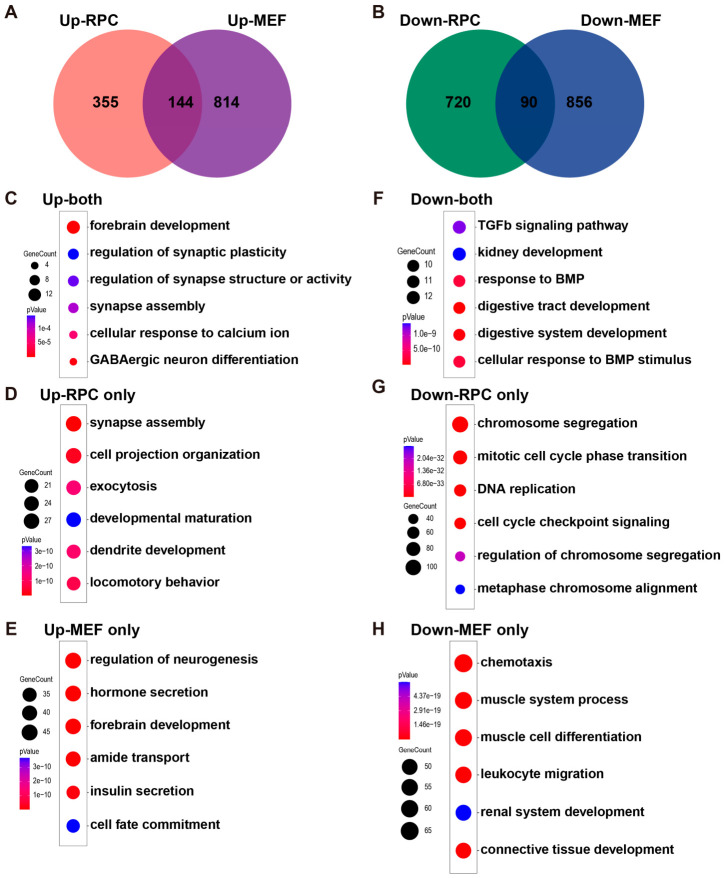
Comparative transcriptome analyses reveal cell-type-specific and conserved gene programs induced by *Ngn3* in late RPCs and MEFs. (**A**,**B**) Venn diagrams showing the overlap of upregulated (**A**) and downregulated (**B**) DEGs between *Ngn3*-OE late RPCs (DAE 2) and *Ngn3*-OE MEFs. (**C**–**H**) Bubble plots showing the top six significantly enriched GO terms for DEGs that are commonly regulated or cell-type-specifically regulated in late RPCs or MEFs: (**C**) GO terms for commonly upregulated genes between late RPCs and MEFs. (**D**) GO terms for late RPC-specifically upregulated genes. (**E**) GO terms for MEF-specifically upregulated genes. (**F**) GO terms for commonly downregulated genes between late RPCs and MEFs. (**G**) GO terms for late RPC-specifically downregulated genes. (**H**) GO terms for MEF-specifically downregulated genes.

Our previous work showed that *Ngn3* potently reprograms distantly related mouse embryonic fibroblasts (MEFs) into RGC-like neurons in vitro, with RNA-seq performed on *Ngn3*-OE MEFs at 1 day post-overexpression to elucidate the underlying mechanisms [25]. This provided a unique opportunity to compare *Ngn3* function in in vivo late RPC reprogramming and in vitro fibroblast reprogramming. Cross-comparison of DEGs between the two cell types revealed a striking cell-type specificity of *Ngn3*-mediated reprogramming pathways, alongside a conserved core neurogenic program. Specifically, among the upregulated genes, only 144 genes were commonly upregulated in both late RPCs and MEFs upon *Ngn3*-OE, whereas 355 genes were uniquely upregulated in late RPCs, and 814 genes were uniquely upregulated in MEFs upon *Ngn3*-OE (Figure 3A). For downregulated genes, merely 90 genes were commonly downregulated in both late RPCs and MEFs upon *Ngn3*-OE, whereas 720 genes were uniquely downregulated in late RPCs and 856 genes uniquely downregulated in MEFs upon *Ngn3*-OE (Figure 3B). These results suggest that *Ngn3* regulates largely distinct gene sets depending on the cellular context in which it is overexpressed. GO term enrichment analyses showed that: the commonly upregulated genes were enriched for cellular processes such as ‘forebrain development’ and ‘synapse assembly’, consistent with the potent neurogenic activity of *Ngn3* across different cell types (Figure 3C); genes uniquely upregulated in late RPCs upon *Ngn3*-OE were further significantly enriched for processes related to neuronal function (Figure 3D), suggesting that the cellular environment in late RPCs was more permissive for the neurogenic function of *Ngn3*; in contrast, genes uniquely upregulated in MEFs upon *Ngn3*-OE were enriched for non-neurogenic pathways such as ‘hormone secretion’ and ‘insulin secretion’, unrelated to neurogenesis and neuronal function (Figure 3E), suggesting that MEFs exhibited multiple potential cell fate options in response to *Ngn3*-OE and required more extensive reprogramming to adopt a neuronal fate. For downregulated genes, the commonly repressed gene set was significantly enriched for cellular process related to TGF-β and BMP signaling pathways (Figure 3F), suggesting that *Ngn3* universally repressed TGF-β superfamily signaling across cell types; genes uniquely downregulated in late RPCs upon *Ngn3*-OE were specifically enriched for processes related to cell cycle progression (Figure 3G), consistent with the strong premature cell cycle exit phenotype observed for *Ngn3*-OE late RPCs (Figure 1), whereas genes uniquely downregulated in MEFs upon *Ngn3*-OE were significantly enriched for cellular processes associated with the mesoderm identity of MEFs, such as ‘muscle system process’ and ‘connective tissue development’ (Figure 3H), suggesting that *Ngn3*-OE significantly repressed the mesoderm identity of MEFs to initiate neuronal reprogramming.

Together, these comparative RNA-seq analyses revealed both conserved and cell-type-specific molecular mechanisms underlying *Ngn3*-driven neuronal fate reprogramming, highlighting the critical impact of the endogenous cellular context on the efficiency and outcome of TF-mediated reprogramming.

### 2.4. Ngn3-OE Alters the Chromatin Accessibility Landscape of Late RPCs

**Figure 4:**
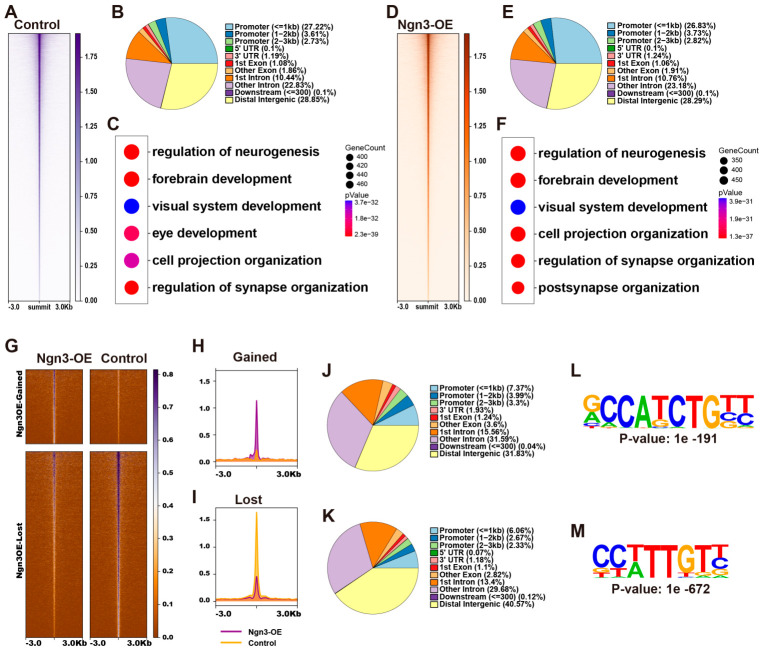
*Ngn3* reshapes the chromatin accessibility landscape of late RPCs. (**A**,**D**) Heatmaps showing accessible genomic regions in control (**A**) and *Ngn3*-OE (**D**) late RPCs. Regions are ordered by signal intensity. (**B**,**E**) Pie charts showing the genomic distribution of accessible chromatin peaks in control (**B**) and *Ngn3*-OE (**E**) late RPCs. (**C**,**F**) Bubble plots showing the top six significantly enriched GO terms for genes associated with open chromatin regions in control (**C**) and *Ngn3*-OE (**F**) late RPCs. (**G**) Heatmaps showing ATAC-seq reads density of DARs induced by *Ngn3*-OE in *Ngn3*-OE and control late RPCs. Regions are ordered by signal intensity. (**H**,**I**) Aggregate profile plots of ATAC-seq signal intensity at gained (**H**) and lost (**I**) DARs in Control vs. *Ngn3*-OE late RPCs. Purple represents the *Ngn3*-OE group, while yellow represents the Control group. (**J**,**K**) Pie charts showing the genomic distribution of gained (**J**) and lost (**K**) accessible chromatin peaks. (**L**,**M**) The most significantly enriched TF binding motif in gained (**L**) and lost (**M**) DARs.

TFs often cooperate with epigenetic regulatory factors to modulate gene expression via epigenetic remodeling. To investigate how *Ngn3*-OE reshaped the epigenetic landscape of late RPCs, we performed ATAC-seq on control and *Ngn3*-OE late RPCs at DAE 2. To this end, we collected GFP-labeled control and *Ngn3*-OE electroporated late RPCs at DAE 2 via FACS and performed ATAC-seq analyses. Calculation of the Fraction of Reads in Peaks (FRiP) showed FRiP scores of 40.73% and 44.02% for the control and *Ngn3*-OE samples, respectively, indicating the high quality of our ATAC-seq dataset. Peak calling identified 128,805 accessible chromatin peaks in control late RPCs (Figure 4A, and Supplementary Material S2). Annotation of the peaks revealed that accessible genomic regions in control late RPCs were predominantly localized to promoter regions and putative distal intergenic and intronic enhancer regions (Figure 4B). GO term enrichment analyses of genes associated with these accessible regions showed significant enrichment for retinal development and function-related processes, such as ‘regulation of neurogenesis’, ‘eye development’, and ‘regulation of synapse organization’ (Figure 4C), consistent with the neural progenitor identity and endogenous developmental potential of late RPCs. For *Ngn3*-OE late RPCs, we identified 136,708 accessible chromatin peaks (Figure 4D, and Supplementary Material S2). Accessible genomic regions in *Ngn3*-OE late RPCs were also predominantly localized to promoter regions and putative distal intergenic and intronic enhancer regions (Figure 4E), and were also enriched for genes involved in retinal development and function (Figure 4F).

We next compared the chromatin accessibility between *Ngn3*-OE and control RPCs to identify differential accessible regions (DARs) induced by *Ngn3*-OE. This analysis identified 2333 genomic regions that gained accessibility and 5842 genomic regions that lost accessibility upon *Ngn3*-OE in late RPCs (Figure 4G–I, and Supplementary Material S2). Annotation of gained and lost genomic regions in *Ngn3*-OE late RPCs revealed that these regions were significantly enriched in putative distal intergenic and intronic enhancer regions, with much smaller proportion localized to promoter regions (Figure 4J,K). This finding suggests that *Ngn3* primarily exerts its epigenetic regulatory effects on enhancers. Next, we searched for consensus DNA sequence motif among DARs upon *Ngn3*-OE in late RPCs. The results revealed that the most prominently enriched consensus DNA motif in genomic regions gained accessibility upon *Ngn3*-OE were 5′-RCCATCTGBY-3′ E-box type motif (Figure 4L), which was identical to the canonical NGN3-binding motif identified by CUT&RUN profiling in pancreatic endocrine progenitor cells [26]. This finding suggests that NGN3 binds directly to its cognate E-box motifs to open chromatin in late RPCs to establish a neurogenically permissive epigenetic landscape. In contrast, the most enriched motifs in genomic regions lost accessibility upon *Ngn3*-OE were those recognized by SOX family homeodomain TFs (Figure 4M), implying that NGN3 indirectly represses chromatin accessibility at these loci via modulation of SOX family TF activity or expression.

### 2.5. Ngn3 Regulates Gene Expression via a Bifurcated Epigenetic Strategy in Late RPCs

**Figure 5:**
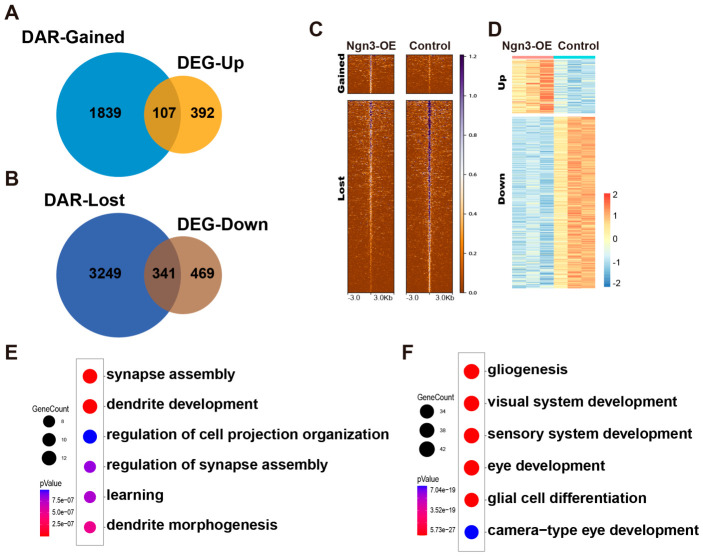
Link between *Ngn3*-induced epigenetic remodeling and transcription changes in late RPCs. (**A**) Venn diagrams showing the overlap between upregulated DEGs and genes associated with gained DARs. (**B**) Venn diagrams showing the overlap between downregulated DEGs and genes associated with lost DARs. (**C**) Heatmaps showing ATAC-seq reads density of DARs associated with the 107 common genes in panel (**A**) (upper, gained) and 341 common genes in panel (**B**) (lower, lost). (**D**) Heatmap showing the RNA-seq expression levels of the same gene set as in panel (**C**,**E**,**F**). Bubble plots showing significantly enriched GO term for the 107 common genes in panel (**A**,**E**) and the 341 common genes in panel (**B**,**F**).

Finally, we investigated the link between *Ngn3*-induced epigenetic remodeling and transcriptomic changes to elucidate the epigenetic regulation of gene transcription upon *Ngn3*-OE in late RPCs. Given that chromatin accessibility is typically positively correlated with transcriptional upregulation, we intersected the list of upregulated genes from RNA-seq with genes associated with gained accessible genomic regions from ATAC-seq. Notably, only a small subset of upregulated genes was associated with increased chromatin accessibility (Figure 5A,C,D), suggesting that *Ngn3* promotes gene transcription in late RPCs without altering chromatin accessibility. By contrast, a much larger proportion of downregulated genes was associated with reduced chromatin accessibility (Figure 5B–D), suggesting that chromatin remodeling is a major mechanism underlying *Ngn3*-mediated gene repression in late RPCs. These results revealed a bifurcated epigenetic regulatory strategy employed by *Ngn3* in promoting and repressing gene expression in late RPCs. GO term enrichment analyses of those genes that were upregulated in *Ngn3*-OE late RPCs and exhibited increased chromatin accessibility were enriched for cellular processes related to neuronal function (Figure 5E), consistent with neurogenic TF character of Ngn family TFs. The downregulated genes that were associated with decreased chromatin accessibility were prominently associated with gliogenesis (Figure 5F), which aligns with the robust repression of Müller glial fate in *Ngn3*-OE late RPCs observed in our previous study [25]. This finding highlights that Ngn3-mediated epigenetic repression via chromatin closure is a key and stable mechanism for inhibiting glial fate in late RPCs. Collectively, these analyses establish a clear epigenetic basis for *Ngn3*-mediated transcriptomic remodeling and differentiation competence reprogramming in late RPCs.

## 3. Discussion

Thorough understanding of the molecular mechanisms governing retinal cell fate development is not only a central question in theoretical biology, but also the foundation for developing innovative biomedical strategies to address the therapeutic challenges faced by ophthalmologists treating retinal degenerative diseases. Accumulating evidence has highlighted the key roles of bHLH family TFs in driving retinal neuronal fate commitment [6]. Among these, *Ngn2* is one of the earliest expressed neurogenic bHLH TFs and orchestrates the leading edge of retinal neuron differentiation [21]. Beyond development and retinas, Ngn family TFs have been shown to possess potent neurogenic capacity in inducing neuronal fate conversion of distantly related somatic cells via direct somatic cell reprogramming [23]. To explore the retinogenic potential of Ngn family TFs, we previously tested the effects of *Ngn1/2/3* overexpression in in vitro cultured MEFs and in vivo electroporated RPCs. This study demonstrated that Ngn family TFs exert robust retinal neuron fate-inducing effects on both lineage-related RPCs and distantly related MEFs. However, the underlying molecular mechanisms, particularly in the in vivo retinal context, remain largely unexplored. In this study, we combined immunohistological examination, RNA-seq and ATAC-seq to dissect the cellular and molecular mechanisms underlying *Ngn3*-mediated reprogramming of the retinogenic competence of late RPCs. Our immunohistological analyses of DAE 2 retinas revealed that *Ngn3*-OE drove late RPCs to undergo premature cell cycle exit. Transcriptomic profiling by RNA-seq demonstrated that *Ngn3*-OE significantly altered the transcriptome of late RPCs, and identified the key molecular mediators underlying the neurogenic and cell cycle exit effects of *Ngn3*-OE in these cells. ATAC-seq analyses uncovered that *Ngn3* regulates gene expression through both epigenetic remodeling-dependent and -independent strategies. Collectively, these findings fill the knowledge gap in Ngn family-mediated retinal cell fate regulation and provide mechanistic insights to optimize Ngn-based in vivo retinal regeneration strategies.

During neurogenesis, cell differentiation and cell cycle progression are tightly coupled, in that a neural progenitor cell must first exit the cell cycle before its terminal differentiation. Consistent with the potent neurogenic activity of Ngn family TFs, in the retina, *Ngn2* expression in early RPCs drives these progenitors to exit the cell cycle and differentiate into RGCs—a process that shapes the formation and movement of the leading front of retinogenesis in the embryonic retina [21]. In this study, we found that *Ngn3*-OE in late RPCs similarly promoted cell cycle exit, despite *Ngn3* not being an endogenously expressed retinogenic TF in the retina. This *Ngn3*-induced premature cell cycle exit likely disrupts the endogenous developmental clock that links proliferation status to lineage bias, which might contribute to the elevated rod-genesis phenotype observed at DAE 14 since postnatal day 3 (DAE 2) represents the peak time of rod neurogenesis. Furthermore, although RNA-seq was performed at DAE 2—well before overt cell fate differentiation phenotypes are detectable—key regulatory and marker genes for the promoted retinal cell fates (e.g., rods and RGCs) and repressed fates (e.g., Müller glia, bipolar cells, and amacrine cells) already exhibited significant transcriptional alterations. This observation suggests that *Ngn3*-induced transcriptomic remodeling is a causal driver of retinal fate reprogramming, rather than a downstream consequence of cell fate commitment. This positions *Ngn3* as a potent regulator that can shift the balance of late RPC fate specification toward therapeutically relevant rod and RGC lineages—two cell types most commonly lost in retinal degenerative diseases such as age-related macular degeneration and glaucoma.

Motif enrichment analyses of chromatin regions with gained accessibility in *Ngn3*-OE late RPCs identified the canonical NGN3-binding E-box motif (5′-RCCATCTGBY-3′) as the most significantly enriched sequence, suggesting that NGN3 may function as a pioneer TF in late RPCs: it directly binds to its cognate motifs to open closed chromatin and establish a pro-retinogenic epigenetic landscape. In contrast, chromatin regions with lost accessibility in *Ngn3*-OE late RPCs were enriched for SOX family TF-binding motifs, pointing to an indirect epigenetic mechanism: *Ngn3* likely represses the expression or activity of Sox family TFs, leading to chromatin closure at their target loci. Sox TFs (e.g., *Sox2*, *Sox9*) are core regulators of RPC proliferation and Müller glial fate development; thus, their indirect repression by *Ngn3* likely contributes to both the premature cell cycle exit phenotype and repression of Müller glial differentiation in late RPCs. On the other hand, integrated analysis of RNA-seq and ATAC-seq data revealed that only a small subset (~12.6%) of upregulated genes is associated with gained chromatin accessibility, whereas a much larger proportion (~38.9%) of downregulated genes links to lost accessibility. This finding is consistent with the neurogenically permissive state of late RPCs, eliminating the need for *Ngn3* to induce *de novo* chromatin opening. For gene repression, however, *Ngn3* relies more on chromatin closure to achieve stable and heritable silencing of non-target fate programs—including cell cycle progression and gliogenesis. Future studies employing techniques such as CUT&RUN or ChIP-seq to characterize NGN3-binding sites in late RPCs would provide further mechanistic insights into how *Ngn3*-OE rewires the differentiation competence of these cells.

A key mechanistic insight from this study is the cell-type specificity of *Ngn3*-mediated reprogramming revealed by cross-comparison of DEGs induced by *Ngn3*-OE in late RPCs and MEFs. While only 144 upregulated and 90 downregulated genes were shared between the two cell types, the conserved core program points to a universal neurogenic mechanism of *Ngn3*: shared upregulated genes were enriched for forebrain development and synapse assembly, and shared downregulated genes targeted the TGF-β/BMP signaling pathways. TGF-β/BMP signaling is a well-characterized negative regulator of neurogenesis, and its universal repression by *Ngn3* across both neural progenitors and somatic cells identifies this pathway as a potential core mediator of *Ngn3*’s neurogenic activity. In contrast, the cell-type-specific DEGs reflect the intrinsic differences between late RPCs and MEFs. Late RPCs exhibited unique downregulation of cell cycle progression genes—supporting the premature cell cycle exit phenotype—and unique upregulation of neuronal function-related genes, reflecting a chromatin and transcriptomic landscape that is inherently conducive to *Ngn3*’s retinogenic activity. MEFs, by contrast, displayed unique downregulation of mesodermal identity genes and unique upregulation of non-neurogenic genes, reflecting the need for *Ngn3* to first erase somatic cell identity and the presence of alternative fate potentials in these cells.

In conclusion, this study elucidated the in vivo cellular and molecular mechanisms underlying *Ngn3*-mediated reprogramming of the retinogenic competence of late RPCs. These findings advance our understanding of Ngn family-mediated retinal cell fate regulation and provide a mechanistic framework for optimizing *Ngn3*-based in vivo retinal regeneration strategies for the treatment of retinal degenerative diseases.

## 4. Materials and Methods

### 4.1. Retinal In Vivo Electroporation (IVE)

All animal studies were performed in accordance with the protocol approved by the Institutional Animal Care and Use Committee of Zhongshan Ophthalmic Center (Z2023035). Retinal injection and electroporation were performed on postnatal day 1 (P1) mouse pups following a published protocol [27]. Briefly, P1 pups were anesthetized via hypothermia by placement on ice for 3 min. The palpebral fissure was gently opened using a 1 mL syringe needle (Winner Medical, Hunan, China), and a small incision was made in the sclera near the corneal-scleral limbus using an insulin needle. Under a Leica M620 F20 stereomicroscope (Leica Biosystems, Nussloch, Germany), 5 μL of purified plasmid DNA (2–5 μg/μL in 1 × PBS) was delivered into the subretinal space using a Hamilton syringe equipped with a 32-gauge blunt-ended needle (Hamilton, Bonaduz, Switzerland). Immediately following injection, tweezer-type electrodes (dipped in 1 × PBS) were placed to strictly encompass the head, with the anode positioned over the injected eye. Five square-wave pulses (80 V; 50 ms duration; 950 ms interval) were applied using a Gemini X2 pulse generator (BTX, Holliston, MA, USA). After the procedure, pups were placed on a warming pad for rewarming and were returned to their mothers once they regained steady physical activity.

### 4.2. Immunohistochemistry and Image Acquisition

Mouse pups were anesthetized via hypothermia on ice at day 2 after electroporation (DAE 2), and the eyes were carefully collected. The harvested eyes were fixed in 4% paraformaldehyde (PFA) at room temperature for 30 min. For cryoprotection, the samples were dehydrated in a graded sucrose series (15% and 30% *w*/*v* in 1 × PBS) until fully equilibrated. Tissues were then embedded in Tissue-Tek O.C.T. compound (Sakura Finetek, Torrance, CA, USA) and cut into 10 μm sections using a Leica CM1950 cryostat microtome (Leica Biosystems, Nussloch, Germany). For immunofluorescence analysis, heat antigen retrieval was performed by incubating the sections in citrate buffer (pH 6.0) at 95 °C for 30 min. After cooling to room temperature, sections were blocked and incubated with primary antibodies diluted in PBST (1 × PBS with 0.1% Triton X-100(Sigma-Aldrich, St. Louis, MO, USA)) supplemented with 5% fetal bovine serum (FBS) overnight at 4 °C. To identify proliferating late retinal progenitor cells (RPCs), a rabbit anti-Ki67 primary antibody (Abcam, Cambridge, UK; ab15580) was employed. Following three washes with PBST, sections were incubated with appropriate fluorophore-conjugated secondary antibodies for 1 h at room temperature. Nuclei were counterstained with 4′,6-diamidino-2-phenylindole (DAPI). Finally, the slides were mounted with cover glasses using VECTASHIELD mounting medium (Vector Labs, Newark, CA, USA). Immunofluorescence images were acquired using a confocal microscope (Zeiss LSM 980; Carl Zeiss Microscopy GmbH, Jena, Germany) and analyzed via imageJ woftware (version 1.53f) [28].

### 4.3. TUNEL Assay

To detect apoptotic cells, Terminal deoxynucleotidyl transferase dUTP nick end labeling (TUNEL) staining was performed on cryosections. Briefly, the TUNEL reaction was prepared by mixing TdT enzyme and fluorescence labeling solution at a 1:9 ratio (*v*/*v*). After washing, cryosections were incubated with the TUNEL reaction mixture for 1 h at 37 °C in the dark. After washing slides were mounted using an anti-fluorescence quenching medium and stored at −20 °C. TUNEL images were acquired using a confocal microscope (Zeiss LSM 980; Carl Zeiss Microscopy GmbH, Jena, Germany) and analyzed via imageJ software (version 1.53f) [28].

### 4.4. Statistical Analysis for Immunofluorescence and TUNEL Analyses

The data of immunofluorescence and TUNEL analyses were performed in at least three independent biological replicates. Data are expressed as mean ± SD, and statistical significance was determined using two-tailed Student’s *t*-tests. A *p*-value threshold of less than 0.05 was defined as statistically significant.

### 4.5. Fluorescence-Activated Cell Sorting (FACS)

For fluorescence-activated cell sorting (FACS), retinas were harvested from pups at DAE 2. Tissues were dissociated into single-cell suspensions using Papain (Worthington Biochemical Corp., Lakewood, NJ, USA) and resuspended in 1 × PBS supplemented with 0.04% bovine serum albumin (BSA). GFP^+^ populations were subsequently isolated using a BD FACSAria Fusioncell sorter (Becton, Dickinson and Company, Franklin Lakes, NJ, USA).

### 4.6. RNA-Seq

Sorted GFP^+^ cells (100,000 cells per replicate) were harvested by centrifugation and resuspended in 200 μL of TRIzol reagent (Beyotime, Shanghai, China), with the volume adjusted according to the retinal cell input. Total RNA was extracted following the manufacturer’s instructions. Sequencing libraries were constructed using the NEBNext Ultra RNA Library Prep Kit for Illumina (New England Biolabs, Ipswich, MA, USA) according to the recommended protocol. The resulting libraries were sequenced on the Illumina NovaSeq X platform to generate 150 bp paired-end reads. The raw sequencing data were processed using Trimmomatic (version 0.39)to remove adapter sequences and filter out low-quality bases, yielding high-quality clean reads. These reads were then mapped to the mouse reference genome (mm10) using HISAT2 (version 2.2.0). Gene-level quantification was performed using Subread (version 2.0.1) to generate raw read counts. Differential expression analysis was conducted using the DESeq2 R package, using a negative binomial model and Wald test for two-group comparisons. Multiple testing correction was conducted using the default Benjamini–Hochberg procedure to control the false discovery rate (FDR). Differentially expressed genes (DEGs) were identified based on a significance threshold of adjusted *p*-value ≤ 0.05 and a log_2_ (fold change) ≥ 0.5/≤ −0.5. Functional annotation and Gene Ontology (GO) enrichment analysis were performed using the clusterProfiler R package. For RNA-seq analysis, three independent biological replicates were performed for both *Ngn3*-OE and control groups.

### 4.7. Assay for Transposases-Accessible Chromatin Using Sequencing (ATAC-Seq)

ATAC-seq libraries were constructed from 5 × 10^4^ FACS-isolated cells per sample using the Hyperactive ATAC-Seq Library Prep Kit for Illumina (TD711; Vazyme, Nanjing, China), following the manufacturer’s instructions. Briefly, nuclei were isolated using a chilled lysis buffer, subjected to Tn5-mediated transposition at 37 °C for 30 min, and purified using VAHTS DNA clean beads. After PCR amplification and indexing, libraries were sequenced on an Illumina NovaSeq X platform (150 bp paired-end). For data analysis, raw reads were trimmed using Trimmomatic and aligned to the mm10 genome via Bowtie2. After removing duplicates (Picard, version 2.20.4), mitochondrial reads (SAMtools, version 1.6), and ENCODE blacklisted regions (bedtools, version 2.30.0), fragments ≤120 bp were extracted. Reads were shifted (+4/−5 bp) using deepTools (version 3.1.3) to account for Tn5 duplication prior to peak calling with MACS2 (version 2.2.4) (q < 0.05). Differentially accessible regions (DARs) were identified using DiffBind (version 3.18.0) combined with DESeq2 with a threshold of log_2_(fold change) > 1 and *p*-value < 0.05. Motif enrichment analysis was performed using HOMER (version 4.11). Fraction of Reads in Peaks(FRiP) was calculated as the ratio of reads mapped within called ATAC-seq peaks to total mapped reads, using featureCounts. A single biological sample was performed for ATAC-seq library construction and sequencing for each experimental group.

## Data Availability

The original data presented in the study are openly available in GEO under at https://www.ncbi.nlm.nih.gov/geo/ (accessed on 30 March 2026) (accession number GSE326387 and GSE326388).

## Supplementary Materials

The following supporting information can be downloaded at: https://www.mdpi.com/article/10.3390/ijms27093845/s1.
